# Quality of life among immigrants in Swedish immigration detention centres: a cross-sectional questionnaire study

**DOI:** 10.3402/gha.v8.28321

**Published:** 2015-07-17

**Authors:** Soorej J. Puthoopparambil, Magdalena Bjerneld, Carina Källestål

**Affiliations:** Department of Women's and Children's Health, International Maternal and Child Health, Uppsala University, Uppsala, Sweden

**Keywords:** quality of life, QOL, immigration detention, immigrant detainee, refused asylum seekers, Sweden

## Abstract

**Background:**

Detention of immigrants negatively affects their health and well-being. Quality of life (QOL) is a broad concept incorporating the self-evaluation of one's own health and well-being that can provide an understanding of the health and well-being of immigrant detainees. The aim of this study was to estimate QOL among immigrant detainees in Sweden and to assess its relationship with the services provided in detention centres and with the duration of detention.

**Design:**

All immigrants in all five existing Swedish detention centres (*N=*193) were invited to participate in the study (n=127). In this cross-sectional study, QOL was measured using the WHOQOL-BREF questionnaire, which was administered by the first author. The questionnaire contained four additional questions measuring participants’ satisfaction with the services provided in detention. Associations between QOL domain scores and service satisfaction scores were assessed using regression analysis. The Spearman's rank correlation coefficient was calculated to measure the degree of association between the duration of detention and QOL scores.

**Results:**

The mean QOL domain scores (out of 100) were 47.0, 57.5, 41.9, and 60.5 for the environmental, physical, psychological, and social domains, respectively. The level of support detainees received from detention staff was significantly positively associated with detainees’ physical (β_adjusted_ 3.93, confidence interval [CI] 0.06–7.80) and psychological (β_adjusted_ 5.72, CI 1.77–9.66) domain scores. There was also significant positive association between detainees’ satisfaction with the care they received from detention staff and the domain scores. The general health score in the WHOQOL-BREF was significantly associated with the detainees’ ability to understand the Swedish or English languages. Although not statistically significant, a longer duration of detention was negatively correlated with QOL scores.

**Conclusion:**

Immigrant detainees report low QOL. Services provided at the centres, especially the support received from detention staff, is positively associated with their QOL. A review of detention guidelines addressing language barriers, staff training, and duration of detention is highly recommended.

Detention of immigrants, also known as administrative detention, is widely practiced by several countries. The European Parliament and the Council of the European Union define *detention* as ‘*confinement of an applicant by a Member State within a particular place, where the applicant is deprived of his or her freedom of movement*’ (1). An immigrant can be detained in order to prevent absconding and/or non-cooperation with authorities in relation to identity verification or deportation process, to determine or verify his/her identity, to prepare and carry out repatriation, to protect public health, and to protect national security (1, 2). According to the international guidelines, immigration detention should be used as a last resort (1, 2). The majority of detainees in the European Union (EU) are immigrants who have applied for international protection or those who have exhausted the legal process of seeking protection and are waiting to be deported from a host country (3).

Although there are differences in legal systems, studies around the world consistently identify the negative health impacts of detention (4–6). Detainees might be entering detention with pre-existing risk factors such as exposure to trauma and torture (5, 7, 8). In addition to this risk, the detention environment can negatively affect the health and well-being of detainees due to factors such as limited access to healthcare services (6, 9, 10), limited availability of information pertaining to their situation (11), and unfavourable staff behaviour (12, 13). Several studies show a high prevalence of mental illness among detainees (4, 5, 7, 14). The negative impact of detention on mental health persists even after release from detention (9, 15). In order to better understand and mitigate this impact, it is important to explore how detention environment and the services provided therein affect the health and well-being of detainees.

The World Health Organization (WHO) defines *quality of life* (QOL) as *‘individuals’ perceptions of their position in life in the context of the culture and value systems in which they live and in relation to their goals, expectations, standards and concerns’* (16). QOL is a broad concept incorporating individuals’ overall sense of well-being, physical health, psychological health, personal beliefs, social relationships, and their relationship to the salient features of their environment (17, 18). Health is one of the main elements of QOL (18, 19), but QOL goes beyond measuring the traditional measures of mortality and morbidity and asks individuals how concerned or satisfied they are with their lives (18, 20). It is subjective rather than objective, since the individual evaluates his/her own QOL (17, 18).

## Immigration detention in Sweden

Sweden is one of the major asylum recipient countries in the world (21). In 2014, the Swedish Migration Agency (SMA) received 81,301 asylum applications. Among the applications where a decision was made, 17.5% were rejected (22). Sweden detains immigrants belonging to categories such as asylum seekers, immigrants whose asylum applications have been rejected, and immigrants involved in a Dublin procedure (Dublin cases) (3). According to the Dublin III Regulation, an asylum seeker can lodge his/her application in only one EU member state, and all decisions regarding the applicant's asylum will be taken by that EU member state, the Dublin member state. Applicants found living or applying for asylum in another EU member state will be returned to the responsible Dublin member state (23). Legal grounds for detaining an immigrant is laid out in the Aliens Act of 2005 (24).

In 2014, a total of 3201 immigrants were detained in Sweden, of which 10% were females (25). Sweden has 255 detention places spread across five detention centres managed by the SMA. These are secure (locked) facilities, where non-uniformed detention staff manage and provide services to detainees. Occasionally, detainees are placed under police custody in prison for one or two nights for practical reasons (such as long distance) during their transport to detention centres. Detainees are provided with food and other basic necessities, a daily allowance of approximately 2.5 euros, and access to the Internet. They have free access to medical care, which cannot be deferred. Detention staff are the detainees’ main point of contact in the centres, and they provide support to detainees in different ways such as playing cards or billiards with them, assisting them in contacting lawyers or the police, booking interpreters, serving food, and so on.

Recently, there has been an increase in the number of immigrants detained in Swedish detention centres (3). Nevertheless, the use of detention in Sweden is considered to be limited (26, 27), and detention standards are considered to be comparatively better than in other EU member states (3, 27, 28). However, no systematic assessments of the effects of detention on detainees’ health and well-being in Sweden have been conducted thus far.

## Objectives

The primary objective of the study was to assess the QOL of immigrants detained in Swedish immigration detention centres. A secondary objective was to assess the relationship between detainees’ QOL scores and services provided in the detention centres, as well as the duration of detention.

The current study is part of a larger project aimed at identifying factors that could mitigate the effect of detention on the health and well-being of immigrants in Swedish immigration detention centres.

## Methods

### Study design, population, and data collection

A cross-sectional survey using the WHOQOL-BREF questionnaire, administered by the first author, was conducted among immigrants detained in all five immigration detention centres existing in Sweden in 2014.

Because detainees come from different countries and the questionnaire does not exist in all of their native languages, the research team decided to use the English and Swedish versions of the questionnaire. Moreover, the first author was only proficient in Swedish and English. If the participant could not understand Swedish or English, authorized telephone interpreters were arranged through private companies. All the interpreters were briefed about the nature of the study, the questionnaire, and the importance of translating questions and participants’ responses word by word (to the greatest extent possible). This methodology was discussed with and approved by the Health Statistics and Health Information Systems at WHO, Geneva, who provided the validated English and Swedish versions of the questionnaire. In order to test the feasibility of administering the questionnaire using telephone interpreters in immigration detention centres, a pilot study was conducted in one of the five detention centres. Data were collected from 13 detainees. The questionnaire and the use of telephone interpreters were found to be feasible. The survey instrument (WHOQOL-BREF+service satisfaction variables) had high internal consistency (Cronbach's alpha=0.90). Data obtained from the pilot study were included in the final analysis since no changes were made to the questionnaire during the pilot phase.

The first author visited and collected data from all five detention centres existing in Sweden. Each visit took 1 to 1.5 weeks. There were no inclusion or exclusion criteria. The first author had full access to detainees within the centres and could therefore invite all detainees present in the centres (*N=*193), individually, to participate in the study. A total of 127 detainees participated in the study. The majority of the detainees who declined to participate reported that this was because they could see no legal benefits in participating or because they were stressed. Seven detainees could not participate because a telephone interpreter was not available at the time. Others declined without giving any reason.

In total, 77 detainees used the help of telephone interpreters to answer the questionnaire. All participants were encouraged to answer the questionnaire by themselves, and 16 participants did so. The first author administered the questionnaire to all other participants. The questionnaire was administered individually to each participant and only the participant and the first author were present during this process.

### Ethical considerations

Ethical approval for the study was obtained from the Regional Ethical Review Board in Uppsala, Sweden. Before administering the questionnaire, all participants were verbally informed about the study, the voluntary nature of their participation, and the absence of any legal or other benefits as a result of their participation. They were also informed that data obtained through the study would be kept confidential and that only the research team would have access to it. All participants were given a copy of an information sheet containing the above-mentioned information and contact information of the research team. No identifying information such as names or identification numbers were collected. Verbal consent was obtained from all participants.

### Survey instrument and variables

The WHOQOL-BREF, a shorter version of the WHOQOL-100, is a 26-item questionnaire developed by the WHO. The questionnaire was developed through field trials conducted in 14 countries using 12 different languages, making it cross-culturally valid and ideal for use in multicultural groups such as immigrant detainees (16, 19, 20). It is one of the most widely used tools for measuring QOL (19). The questionnaire contains 24 questions measuring QOL scores in four domains—environmental (eight questions), physical (seven questions), psychological (six questions), and social (three questions); in addition to two questions measuring general QOL and health (17). The questionnaire can be either self-administered or interview-administered (17).

In addition to the WHOQOL-BREF questions, participants were asked four questions that measured their satisfaction with the services provided at the centres (service satisfaction scores). These questions were developed from qualitative studies conducted by the authors (13, 29). Table 1 shows the main outcome variables and their scoring scale. Data on sociodemographic characteristics, as well as attributes that were specific to life in detention, were collected. *Educational level* was defined as *primary* (5 years of schooling), *secondary* (12 years of schooling), or *tertiary* (education occurring after secondary-level education). Responses to questions that asked whether they were currently or previously ill and required medical treatment were based on their own judgement. *Legal status* was defined as follows: *asylum seeker* (an individual whose asylum application had not yet received a final decision); *refused asylum seeker* (an individual who was not granted asylum); *Dublin case* (an individual subject to the Dublin Regulation), and *irregular migrant* (an individual who did not belong to any of the above categories and did not possess a valid permit to stay in the country).

**Table 1:** QOL and service satisfaction scoring scale

WHOQOL-BREF	Scoring scale
Environmental domain	0–100
Physical domain	
Psychological domain	
Social domain	
General QOL in detention	1–5
	1: Very poor
	5: Very good
General health	1–5
	1: Very dissatisfied
	5: Very satisfied
Service satisfaction scores (scale)^a^	
Level of support received from detention	1–5
staff	1: Not at all
	5: Completely
Ability to understand information	1–5
provided by authorities	1: Not at all
	5: Completely
Satisfaction with care provided by	1–5
detention staff	1: Very dissatisfied
	5: Very satisfied
Satisfaction with food provided	1–5
	1: Very dissatisfied
	5: Very satisfied

a These variables were added to the WHOQOL-BREF to capture detainees’ satisfaction on services provided at the detention centres. *Note*: QOL, quality of life.

In order to assess the association between the service satisfaction variables and the QOL domain scores, the four service satisfaction scores were considered explanatory variables and the four QOL domain scores were considered outcome variables. Age, gender, educational level, partner living in Sweden, child(ren) living in Sweden, and duration of participants’ stay in Sweden (excluding time spent in detention) were considered potentially confounding sociodemographic factors. The participants’ legal status, the detention unit where they were being detained, the duration of detention, and their knowledge of their date of departure from Sweden were considered potentially confounding detention-related factors.

### Statistical analysis

QOL scores were calculated as per the instructions provided in the WHOQOL user manual (17). The scores of all items within each domain were added to get raw WHOQOL-BREF domain scores, which were then transformed to a 0–100 scale. If a response for one item was missing, it was substituted with the mean of the other scores in that domain. A maximum of two missing responses was allowed for the score to be calculated in all domains except for the social domain. If more than one item response was missing in the social domain, the domain score was not calculated.

WHOQOL-BREF domain scores were treated as continuous numerical variables (on a scale of 0–100). The general QOL and health scores and the service satisfaction scores were treated as categorical/ordinal variables (on a scale of 1–5). Descriptive statistics were obtained through frequency analysis. A chi-square test was used to evaluate the association between categorical variables.

Simple and multiple linear regression analyses were performed to assess the association between the QOL domain scores and the service satisfaction scores. Assumptions underlying the linear regression were checked by plotting *x* (service satisfaction scores) against *y* (QOL domain scores) to assess the linear relationship between them. The assumption for normal distribution of *y* was checked by inspecting histograms. Two models were used to perform multiple linear regression analysis. The first model assessed the relationship between the explanatory (service satisfaction scores) and outcome variables (QOL domain scores), adjusting for potential confounding sociodemographic factors. Because the focus of the study was on the services provided at the detention centres, in addition to the sociodemographic factors (Model 1), potential confounding factors related to the detention centres were included in the second model. The Spearman's rank correlation coefficient was calculated to estimate the degree of association of duration of detention (numerical variable) with duration of stay in Sweden, service satisfaction scores, general QOL, general health (categorical variables), and the domain scores (numerical variables). The Spearman's rank correlation coefficient was chosen because of the non-linearity between the duration of detention and other variables. Neither duration of detention nor stay in Sweden were normally distributed.

A plot with a smooth curve fitted using locally weighted scatterplot smoothing was used to visualize the relationship between duration of detention and psychological domain score. A value of *p*<0.05 was considered statistically significant. All statistical analyses were performed using the software program R (30).

## Results

### General characteristics of the study sample

Table 2 displays the sociodemographic characteristics of the participants. They were mainly males (93%), and approximately 43% were in the 20–30-year age group. Approximately 34% had their partners living in Sweden, 17% had their children living in Sweden, and around 12% had lived in Sweden for more than 4 years. There were 46 different nationalities, with Albanians (9%), Georgians (8%), Afghans (7%), and Algerians (6%) being the most common groups. Table 3 provides the frequency distributions of characteristics that are specific to detention. The majority of the participants were refused asylum seekers (68.5%), and the average duration of detention was 37.8 days (SD=57.3). More than half of the participants considered themselves ill and in need of medical care.

**Table 2:** Sociodemographic characteristics of the detainees in Swedish detention centres

	Frequency (%)
Gender	
Male	118 (92.9)
Female	9 (7.1)
Age groups in years (17–60 years)	
≤20	17 (13.4)
>20–≤30	55 (43.3)
>30–≤40	40 (31.5)
>40–≤50	11 (8.7)
>50	4 (3.1)
Nationality (top five)^a^	
Albania	11 (8.7)
Georgia	10 (7.9)
Afghanistan	9 (7.1)
Algeria	8 (6.3)
Nigeria	6 (4.7)
Educational level^b^	
None	16 (12.6)
Primary	36 (28.3)
Secondary	46 (36.3)
Tertiary	29 (22.8)
Marital status	
Single/widowed/divorced	64 (50.4)
Married/cohabiting/in a relationship	63 (49.6)
Partner living in Sweden	43 (33.9)
Child(ren) living in Sweden	21 (16.5)
Duration of stay in Sweden in years	
(0–20 years)	
≤1	45 (35.4)
>1–≤2	25 (19.7)
>2–≤3	25 (19.7)
>3–≤4	17 (13.4)
>4	15 (11.8)

a There were 46 different nationalities present among detainees.

b *Primary*: 5 years of schooling; *Secondary*: 12 years of schooling; *Tertiary*: education occurring after secondary-level education.

**Table 3:** Background characteristics specific to the detainees in Swedish detention centres

	Frequency (%)
Number of participants (response rate)	127/193 (65.8)
Åstorp detention centre	34 (26.8)
Flen detention centre	35 (27.6)
Gävle detention centre	17 (13.4)
Kållered detention centre	16 (12.6)
Märsta detention centre	25 (19.7)
Legal status^a^	
Asylum seeker	2 (1.6)
Refused asylum seeker	87 (68.5)
Dublin case	29 (22.8)
Irregular migrant	9 (7.1)
Has information about departure date	15 (11.8)
Duration of detention (1–270 days)	
Mean	37.8 days (SD=57.3)
Placed in a prison while being transported to detention	63 (49.6)
Worked in Sweden before being detained	43 (33.9)
Ill before being detained^b^	53 (41.7)
Currently ill^b^	68 (53.4)

a *Asylum seeker*: an individual who has not yet received a final decision on their asylum application; *Refused asylum seeker*: an individual who is not granted asylum; *Dublin case*: an individual subject to the Dublin procedure; *Irregular migrant*: an individual who does not belong to any of the above categories and does not possess a valid permit to stay in the country.

b As defined by the participant.

### QOL and service satisfaction scores

Table 4 shows the QOL and service satisfaction scores. The psychological domain had the lowest mean score, 41.9 (SD=19.3). General health and QOL in detention were given median scores of two out of five. Detainees were moderately satisfied (giving a score of three out of five) with the services provided at the centres. There was no significant difference in QOL and service satisfaction scores between the detention centres. All service satisfaction scores were significantly associated with each other (results not shown).

**Table 4:** WHOQOL-BREF and service satisfaction scores

WHOQOL-BREF score (scale)	Mean score (SD)
Environmental domain (0–100)	47.0 (16.3)
Physical domain (0–100)	57.5 (18.4)
Psychological domain (0–100)	41.9 (19.3)
Social domain (0–100)	60.5 (19.9)
	Median score (IQR)
General QOL in detention (1–5)	2 (1–3)
General health (1–5)	2 (2–4)
Service satisfaction scores (scale)	Median score (IQR)
Level of support received from detention staff (1–5)	3 (2–4)
Ability to understand information provided by authorities (1–5)	3 (2–4)
Satisfaction with care provided by detention staff (1–5)	4 (3–4)
Satisfaction with food provided (1–5)	3 (2–4)

*Note*: QOL, quality of life; IQR, interquartile range.

The general QOL score was not significantly associated with the service satisfaction scores. However, the general health score was significantly associated with the level of support received from detention staff (Fisher's exact test; *p*=0.02) and participants’ satisfaction with the care received (Fisher's exact test; *p*=0.02). Table 5 shows the association between the service satisfaction scores and the QOL domain scores. The majority of service satisfaction scores were significantly positively associated with the QOL domain scores without adjusting for possible confounding factors. This positive association was further evident through Spearman's rank correlation coefficient (results not shown). After adjusting for the potentially confounding sociodemographic factors (Model 1), the majority of associations remained statistically significant. Model 2 included the sociodemographic factors from Model 1 and the potential confounding factors specific to detention. The positive association between participants’ satisfaction with care and physical (β_Model2_=6.69, confidence interval, CI [95%] 2.02–11.36), psychological (β_Model2_=5.76, CI [95%] 0.69–10.83), and environmental (β_Model2_=4.20, CI [95%] 0.29–8.17) domain scores remained significant. The same was true for the association between the level of support received by the participants and physical (β_Model2_=3.93, CI [95%] 0.06–7.80), psychological (β_Model2_=5.72, CI [95%] 1.77–9.66) and social (β_Model2_=4.59, CI [95%] 0.64–8.54) domain scores.

**Table 5:** Association between service satisfaction scores and WHOQOL-BREF domain scores

	Physical domain			Psychological domain			Social domain			Environmental domain		
				
	Unadjusted β CI (95%)	Model 1 β CI (95%)	Model 2 β CI (95%)	Unadjusted β CI (95%)	Model 1 β CI (95%)	Model 2 β CI (95%)	Unadjusted β CI (95%)	Model 1 β CI (95%)	Model 2 β CI (95%)	Unadjusted β CI (95%)	Model 1 β CI (95%)	Model 2 β CI (95%)
Level of support received from detention staff	4.07** (1.5–6.7)	4.41* (0.91–7.90)	3.93* (0.06–7.80)	5.90** (3.27–8.54)	6.25** (2.49–10.00)	5.72** (1.77–9.66)	4.63** (1.84–7.43)	4.96** (1.26–8.66)	4.59* (0.64–8.54)	4.78** (2.55–7.02)	3.24* (0.26–6.22)	2.92 (−0.25–6.09)
Ability to understand information provided by authorities	3.80* (0.92–6.66)	3.82 (−0.41–8.06)	3.46 (−1.19–8.11)	5.03** (2.08–7.99)	3.46 (−1.28–8.20)	2.10 (−2.89–7.10)	2.06 (−1.11–5.23)	1.32 (−3.32–5.97)	0.21 (−4.75–5.18)	6.12** (3.74–8.49)	4.67** (1.22–8.13)	3.71 (−0.02–7.44)
Satisfaction with care provided by detention staff	5.37** (2.59–8.14)	6.01** (1.80–10.23)	6.69** (2.02–11.36)	6.07** (3.19–8.94)	4.75 (−0.07–9.58)	5.76* (0.69–10.83)	3.18* (0.05–6.28)	1.29 (−3.51–6.08)	.98 (−4.25–6.22)	6.49** (4.17–8.82)	3.76* (0.11–7.41)	4.20* (0.29–8.17)
Satisfaction with food provided	2.98* (0.09–5.87)	3.96 (−0.17–8.10)	3.90 (−0.71–8.51)	2.26 (−0.79–5.31)	1.23 (−3.48–5.94)	0.74 (−4.27–5.75)	0.84 (−2.32–4.00)	1.05 (−3.50–5.61)	.91 (−5.93–3.97)	4.25** (1.76–6.74)	4.06* (0.63–7.49)	2.60 (−1.19–6.39)

*Notes*: β, regression coefficient; CI, confidence interval. *Model 1*: Adjusted for sociodemographic factors: age, gender, education, partner living in Sweden, child(ren) living in Sweden, duration of stay in Sweden. *Model 2*: Adjusted for sociodemographic and detention characteristics: age, gender, education, partner living in Sweden, child(ren) living in Sweden, duration of stay in Sweden, detention unit, aware of departure date, detention duration, legal status.

* *p*<0.05

** *p*<0.01.

Additionally, the general health score was significantly associated with the participants’ legal status (χ^2^=28.6, df=12, *p*=0.02) and their ability to understand (speak, read) Swedish or English (χ^2^=16.5, df=4, *p*=0.002). The direction of the association with legal status was inconclusive due to the uneven distribution of participants in different legal status categories (see Table 3). There was a positive association between the ability to understand Swedish or English and the general health score.

### Duration of detention and QOL

**Fig. 1:**
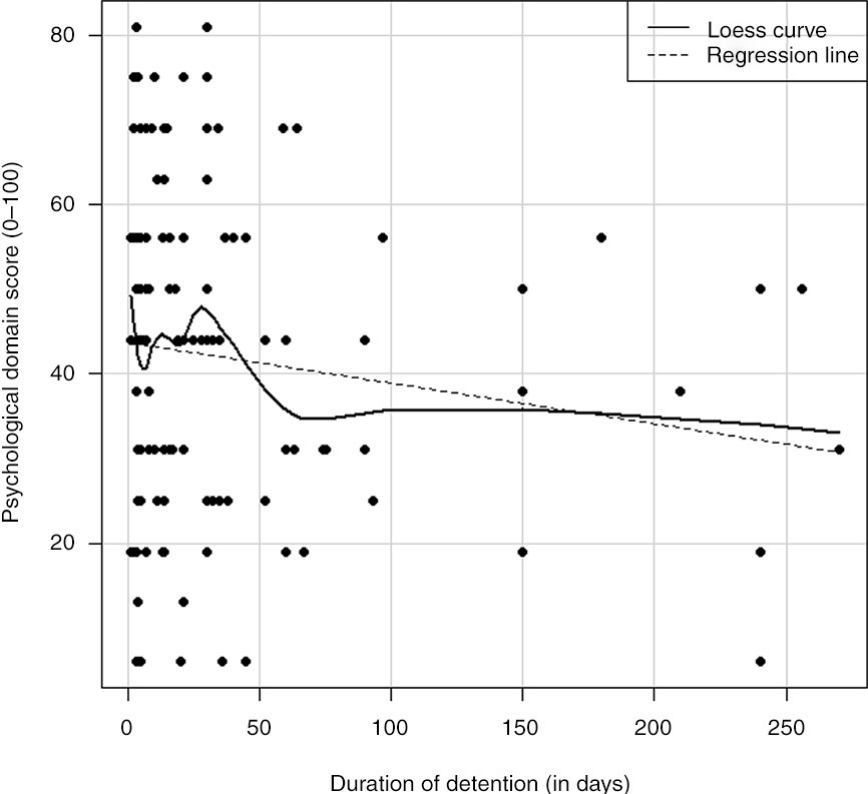
Plot showing the relationship between psychological health and duration of detention.

The negative correlations between the duration of detention and the physical (ρ_s_=−0.11, *p*>0.05), psychological (ρ_s_=−0.11, *p*>0.05), social (ρ_s_=−0.03, *p*>0.05), environmental (ρ_s_=−0.1, *p*>0.05) and general health scores (ρ_s_=−0.14, *p*>0.05), were not statistically significant. However, Fig. 1 shows a fluctuating trend in the detainees’ psychological domain score during the initial period of detention (up to approximately 30 days of detention) followed by a decreasing trend. Physical domain scores also follow a similar trend. The general QOL was significantly negatively correlated to duration of detention (ρ_s_=−0.19, *p*<0.05). Moreover, all service satisfaction scores, except for one, were significantly negatively correlated with the duration of detention. There was almost no correlation between duration of detention and participants’ score on their ability to understand information provided by authorities (ρ_s_=−0.03, *p*>0.05).

Duration of participants’ stay in Sweden was positively correlated with their duration of stay in detention (ρ_s_=0.42, *p*<0.0001).

## Discussion

The results show low QOL (with scores of less than 50 out of 100 on two domain scores and less than three out of five on the general QOL and health scores) among immigrant detainees in Sweden. After adjusting for potential confounders, the level of support detainees receive from detention staff and their satisfaction with the care received were the major explanatory factors associated with their physical, psychological, social, and environmental domain scores. Other factors associated with the detainees’ QOL were the duration of detention and language barrier. This suggests that, irrespective of detainees’ background characteristics, the services provided by detention staff affect detainees’ QOL. Thus, if improved, these services have the potential to mitigate the negative effects of detention on the health and well-being of detainees.

Detention staff constitutes a major part of the detention environment (13). The impact of staff behaviour on detainee health and well-being has been discussed in earlier studies (9–13, 29). Staff practices such as calling detainees by numbers instead of names, threatening detainees, and treating them disrespectfully has been shown to have a negative impact on detainees’ health (11, 29). Our results show a positive association between the increasing level of help provided by detention staff as well as the detainees’ satisfaction of care they received with detainees’ QOL scores.

International and EU regulations require states to provide information that can be understood or is reasonably expected to be understood by detainees (1, 2). However, our study results suggest that detainees were only able to understand just over half of the information provided to them (scoring three out of five on their ability to understand information provided by the authorities). Considering the participants’ life situations and the importance of understanding legal decisions and information, it is important to ensure that detainees have a better understanding of the information provided to them. In Sweden, written legal decisions concerning their case are provided in Swedish. Detention staff, police officers, or lawyers verbally translate this information for detainees, most often using interpreters. In a study conducted among ex-detainees in Australia, 95% of the participants reported that language barriers in detention cause very serious stress (31). Studies conducted in the United Kingdom indicate language difficulties as a reason for detainees’ limited understanding of their situation, their limited capacity to express themselves, and their limited access to services (10, 32).

Living in a host country in legal limbo (e.g. having temporary protection status or being an asylum seeker) has been shown to have negative effects on the health and QOL of immigrants (33–36). None of our study participants had permits to stay in Sweden and 11 of them had been in Sweden for more than 5 years. In our study, the duration of stay in Sweden was positively correlated with the duration of detention. Results from other studies have shown a negative association between increasing duration of detention and mental health (4, 11, 14, 15, 37). This association suggests that ensuring better services or detention conditions might have limited or no impact on detainees’ health and QOL as their duration of detention increases. To mitigate this problem, when immigrants are detained for longer periods, various alternatives to detention such as community supervision or electronic monitoring should be explored (3, 26). In Sweden, reporting to authorities (the SMA or the police) regularly is currently offered as an alternative to detention for those immigrants whom the authorities consider to have a lower risk of absconding and a higher likelihood of cooperating with repatriation. The negative association between QOL scores and duration of detention in our study was not statistically significant, although there was a decreasing trend. Irrespective of the statistical significance, increasing duration of detention is negatively correlated with QOL.

The lack of a significant linear relationship between the duration of detention and QOL scores could be a result of the small sample size. A further reason for this may be the differences in the asylum seeking and reception process in Sweden compared to other countries attributing to a more complex interaction between duration of detention and QOL. Another plausible explanation could be that the duration of detention has a direct impact on mental health, and the WHOQOL-BREF might be less sensitive to these effects compared with instruments such as the Harvard Trauma Questionnaire (38) or the Hopkins Symptom Checklist-25 (39), which are specifically designed to identify mental health issues. All of the studies mentioned above that found significant association between detention duration and mental health used such instruments.

The WHOQOL-BREF does not have a cut-off or reference value (17). Because no studies have been conducted among immigrant detainees assessing their QOL using the WHOQOL-BREF, it is not possible to compare the QOL scores with other studies. However, WHOQOL-BREF scores from a recently conducted Swedish study among immigrants might provide a better understanding of QOL among our population of interest (36). Participants in the study received their residence permits within 3 months prior to baseline assessment. The study reported mean baseline WHOQOL-BREF scores of 72.8, 74.7, 75.9, and 67.7 for the physical, psychological, social, and environmental domains, respectively. The QOL scores reported among our study participants were much lower. It should be noted that QOL is influenced by several factors, including the legal status of participants. All participants in the study mentioned above had residence permits to stay in Sweden, whereas in our study all participants were detained. Nevertheless, this provides an indication of the negative impact of detention on QOL, since it was conducted among immigrants who had been recent asylum seekers.

### Methodological limitations

Due to the cross-sectional nature of the study, causality cannot be inferred. As discussed earlier, Swedish detention context is different from other national contexts, and hence generalizability of the results is limited. Regardless of the context, all studies show a negative impact of detention on the health and well-being of detainees. The WHOQOL-BREF has been used among different types of immigrants such as refugees (35), ex-detainees (9), asylum seekers (33), and other categories of immigrants (36). However, the instrument has not been used to assess QOL among immigrant detainees while they are being detained. We found the WHOQOL-BREF to be a valid and relevant instrument for assessing QOL among detainees. However, 21 and 52 participants, respectively, skipped questions on their satisfaction with their sex life and transportation arrangements in detention. These aspects might be of less relevance to detainees while in detention. In addition, the question on sex life might have been sensitive for some participants. In future, the use of the WHOQOL-BREF in immigration detention centres should be undertaken with this in mind. All participants were repeatedly informed about the absence of any benefit to their legal case as a result of participation in the study, yet the likelihood of participants exaggerating their responses and expecting help cannot be completely ruled out. Participants had previously tried to be free from detention, but none of these attempts had resulted in release. Thus, given no direct benefits, we consider the chances that survey responses were exaggerated to be minimal.

The use of telephone interpreters might have influenced the results. However, the research team decided to use this strategy in order to achieve maximum participation. All practical precautions were taken to minimize the impact of interpreters on participant responses. The first author conducted all the interviews and briefed the interpreters about the nature of the task before starting every interview. The use of telephone interpreters was discussed with and approved by the WHO office in Geneva.

The fact that almost 44% of the detainees chose not to participate might have affected the results, but the extent to which this affected the validity of the results is difficult to ascertain. It was not possible to conduct any analysis of the non-participants, since the research team did not have access to their records. Considering the highly stressful situation in detention and detainees’ strong urge to get out of detention, it is logical that some of them did not want to participate in a study that could not offer them any legal help.

Irrespective of these limitations, the study is important. It is the first study of its kind to assess the QOL of immigrants in detention in Sweden and to explore the association between services provided at the centres and detainees’ QOL.

## Conclusion

Even in a country like Sweden, which is considered to have better detention standards, immigrant detainees have low QOL. As recommended by various international guidelines, detention of immigrants should be used as a last resort. If detained, the duration of detention should be as short as possible, and ways of mitigating the negative effects of detention on the health and well-being of detainees should be thoroughly explored. The findings of this study highlight the need for mitigation efforts in immigration detention centres to aim at minimizing language barriers and in improving staff support and training to provide better services to detainees.
